# Optimized protocol for high-throughput vernalization with speed breeding in winter wheat

**DOI:** 10.1186/s13007-025-01473-7

**Published:** 2025-12-01

**Authors:** Rishap Dhakal, Pablo Sandro, Lucía Gutiérrez

**Affiliations:** 1 Department of Plant and Agroecosystem Sciences, University of Wisconsin-Madison, 1575 Linden Drive, Madison, WI 53706 USA; 2 Department of Plant Breeding, Swedish University of Agricultural Sciences (SLU), Alnarp, SE-230 53 Sweden

**Keywords:** Speed breeding, High-throughput vernalization, Shallow planting, Winter wheat

## Abstract

**Background:**

Wheat ranks third among cereal crops in terms of global production, and its demand is expected to increase as the human population grows. Plant breeding can increase crop production without burdening natural resources, and one way to accelerate genetic gain is through shortening breeding cycles with speed breeding (SB). Speed breeding protocols for winter wheat have been adapted by adding a vernalization phase to existing spring wheat protocols. Although a protocol for the vernalization phase was previously developed, it was not tested for genotypes grown in the Midwest US, which may have higher vernalization requirements. The transition from vegetative to reproductive stages in winter wheat depends mainly on photoperiod, vernalization temperature, and vernalization length, which determines the time needed to reach flowering. Optimizing vernalization under SB in a greenhouse setting is important for applications in breeding programs. Our objectives were to develop a speed breeding protocol for winter wheat that meets the vernalization requirements of all genotypes and to evaluate the interaction between vernalization temperature and sowing depth.

**Results:**

A significant reduction in the time to flowering via speed breeding was achieved. Compared with normal vernalization, high-throughput vernalization adds on average ten days to the time to harvest. A shallow planting depth results in maturity five days earlier than a deep planting depth.

**Conclusions:**

A combination of speed breeding, shallow planting, and high-throughput vernalization will shorten the breeding cycle by 22 days per generation or 44 days per year compared to normal greenhouse conditions. This system is suitable for genotypes with high vernalization requirements and can be combined with high-throughput systems.

**Supplementary Information:**

The online version contains supplementary material available at 10.1186/s13007-025-01473-7.

## Background

Wheat (*Triticum aestivum* L.) is the third most planted cereal in the world [1]. With the increasing global human population, the demand for wheat is expected to increase [2], urging the need to improve farm yields [3, 4]. Moreover, the challenges of unpredictable climate change necessitate the development of cultivars that combine climate adaptation and high genetic yield [5, 6]. To achieve a quick response to new crop challenges, a rapid increase in genetic gain is needed [7]. In wheat breeding schemes, after the initial crosses are made, several generations of inbreeding are needed before the genotypes are evaluated in the field [8]. Methods such asdoubled haploids [9], shuttle breeding [10], single-seed descent [11], and speed breeding [12, 13] have been used to shorten breeding cycles. Speed breeding (SB) is a method that allows rapid generational advancement by accelerating the growth and development of plants through the manipulation of temperature and photoperiod [13, 14]. In wheat, the transition from vegetative to reproductive stages and the length of the cycle to reach flowering are determined mainly by vernalization (i.e. the plants requirement of exposure to a period of low temperatures to induce flowering) and photoperiod [15]. According to the requirements of the genotypes for vernalization, wheat has been classified into winter types (i.e., it requires vernalization) or spring types (i.e., it does not require vernalization) [16]. Similarly, in terms of photoperiod, there are photoperiod-sensitive and photoperiod-insensitive types [17]. Given that photoperiod and vernalization are governed by gene systems with pleiotropic effects and interactions in response to environmental conditions [18], wheat germplasm has diverse photoperiod and vernalization temperature requirements.

The SB protocols for spring wheat [12, 13] have been adapted to winter wheat by adding a vernalization phase [19]. A temperature between 0 and 8 °C is considered an optimum vernalization temperature for winter wheat [20], with some modern wheat genotypes meeting vernalization requirements with temperatures between 10 and 15 °C [21]. Experiments on vernalization optimization in winter wheat have shown that the use of 10 °C and extended photoperiods (22 h) can be effective [19]. However, Cha et al., 2022 [19] were not able to effectively fulfill the vernalization requirements of all winter wheat genotypes (that had high vernalization requirements) with the 10 °C and extended photoperiods (22 h) conditions. In terms of duration, the effective vernalization period is reported to be between 30 and 45 days for most genotypes [22]. Because vernalization requirements and responses vary between genotypes, vernalization protocols require testing of the applicability of this system in diverse genetic backgrounds, such as those used in breeding programs [19, 23]. Using a vernalization temperature of 10 °C eliminates the limitation of specialized facilities to apply the vernalization treatment. Therefore, it allows for the use of simple climate-controlled greenhouses to vernalize large breeding populations in high-throughput systems. In addition to optimizing the vernalization phase, other strategies, such as adjusting plant density [24], regulating soil moisture [25], and micronutrient or growth hormone application [26], could also be employed to accelerate the crop cycle.

Another factor affecting the length of the cycle is sowing depth, which impacts the time to emergence [27] and plant development [28]. Sowing depth affects the seed water contents and temperature, this impacts the three main phases of the germination process (i.e., germination initiation, subcrown development, coleoptile and first leaf formation) [27, 28]. Sowing shallower than 2 cm shortens the time between sowing and emergence by reducing the hypocotyl and subcrown requirements for elongation, resulting in faster emergence [27, 28], which makes shallow sowing a promising strategy to accelerate the plant growth cycle under SB conditions. The goals of our study were to evaluate the interaction between growth systems, vernalization temperature, and planting depth on a set of diverse winter wheat genotypes. Although these factors had been studied before, to our knowledge, no study has evaluated their interactions. Our hypothesis was that planting depth could interact with the vernalization treatment, allowing shallow planted individuals to fulfill the vernalization requirements faster or at higher temperatures. This had not been demonstrated before. Therefore, this study aimed to optimize speed breeding protocols for winter wheat by evaluating the interaction between vernalization temperature and sowing depth.

## Methods

### Plant materials

We used twelve hard red winter wheat genotypes with a set of diverse backgrounds (Table S1). The genotype set included five widely recognized cultivars developed by different breeding programs across the United States, representing diverse latitudes and longitudes adaptation. In addition, four advanced pure lines from the University of Wisconsin wheat breeding program, developed from crosses involving European-origin lines, and three early breeding lines derived from crosses among the University of Wisconsin pure lines were included. The early breeding lines were specifically chosen because some level of heterozygosity was anticipated creating the potential for heterosis for traits of interest. Their inclusion was important to evaluate whether the developed protocol could reliably induce flowering in these lines, as well as in other early breeding lines that may be developed in the future.

### Growing conditions

The experiment was conducted between December 2022 and July 2023 at the Walnut Street Greenhouse of the University of Wisconsin–Madison via a split-split-split plot experimental design with a factorial arrangement of the treatments and four replications (i.e., four replications of each genotype for each treatment combination, Sup. Figure 1). Seeds of each genotype were sown at two depths : shallow planting (SP) at a depth of 1 cm and deep planting (DP) at a depth of 2.5 cm. We used Pro-Mix HP Biofungicide + Mycorrhizae (Premier Tech, Québec, Canada) as the growing medium for the experiment. After emergence, the plants were subjected to two vernalizing treatments for four weeks. For the normal vernalization (NTV) treatment, the plants were maintained at 4 °C with a 16-hour light and 8-hour dark photoperiod using a Kelvinator refrigerated cabinet system with a light intensity of 100–150 µmol m^− 2^ s^− 1^ using fluorescent light. For the high-throughput vernalization (HTV) treatment, the plants were kept at 10 °C with a 22-hour light and 2-hour dark photoperiod in a greenhouse room with a light intensity of 400–500 µmol m^− 2^ s^− 1^ using high pressure sodium vapor lights following previous methods [19]. A multicell tray with 2.5 (length) × 2.5 (width) × 3.5 (depth) cm cells was used at the initial growth stages. At Zadok growth stage 23 [29], the plants were transplanted into 3.8 cm³ pots, with one plant per pot serving as the experimental unit.

After the vernalization period, the plants were moved to two separate greenhouses: one under normal growth conditions (NB; 16-hour photoperiod, 21 °C during the day and 12 °C at night) and one under speed breeding conditions (SB; 22-hour photoperiod, 20 °C during the day and 16 °C at night). In both systems, a light intensity of 400–500 µmol m^− 2^ s^− 1^ was maintained using high pressure sodium vapor lights, and the plants were watered from planting to physiological maturity with adequate water levels. Each pot was fertilized with 26.7 g of Osmocote Smart-Release Plant Food Plus (Scotts Miracle-Gro Company, Marysville, OH). The nutrient content of the fertilizer was 15% nitrogen, 9% available phosphate, 12% soluble potash, 6% sulfur, 1.3% magnesium, 0.46% iron, 0.05% manganese, 0.05% zinc, 0.02% boron, and 0.02% molybdenum.

### Genotyping

Leaf tissue was collected at Zadok 23 and sent to the USDA-ARS Small Grains Genotyping Laboratory, Fargo, ND for genotyping. DNA isolation and library preparation was done following the same procedure as described in Bazzer et al., 2025 [30]. The genotypes were genotyped via a genome-wide Illumina Infinium XT genotyping array that enables the integration of custom multispecies variants. The USDA SoyWheOatBar-3k array contains informative SNPs for soybean, wheat, oat, and barley. For each crop, 3000 SNPs were built. The array was developed by the USDA-ARS Small Grains Genotyping Laboratory, Fargo, ND.

### Plant measurements

The growth stages were recorded following the Zadoks scale [29]. For each pot, the number of days to reach the following Zadok growth stage (ZGS) wasrecorded: three leaves (ZGS 13), three tillers (ZGS 23), first node (ZGS 31), heading (ZGS 59), and physiological maturity (ZGS 87). Individual spikes from the main tillers were harvested after the plants reached ZGS 87 (physiological maturity). The harvested spikes were dried at room temperature in a container with silica gel for three days. Each spike was then threshed using a single-head thresher, and the number of seeds was counted.

### Statistical analysis

The following mixed model was used to obtain the best linear unbiased estimates (BLUE) for all the traits:

1 $$\begin{gathered} {{\mathrm{y}}_{{\mathrm{ijklm}}}}\,=\,\mu \,+\,{{\mathrm{S}}_{\mathrm{i}}}+{\text{ }}{{\mathrm{V}}_{\mathrm{j}}}\,+\,{{\mathrm{b}}_{{\mathrm{k}}\left( {{\mathrm{ij}}} \right)}}\,+\,{{\mathrm{D}}_{\mathrm{l}}}\,+\,{{\delta}}*{\text{ }}+{\text{ }}{{\mathrm{G}}_{\mathrm{m}}}+{\text{ S}}{{\mathrm{V}}_{{\mathrm{ij}}}}+{\text{ S}}{{\mathrm{D}}_{{\mathrm{il}}}}+{\text{ V}}{{\mathrm{D}}_{{\mathrm{jl}}}}+ \hfill \\ {\mathrm{S}}{{\mathrm{G}}_{{\mathrm{im}}}}+{\mathrm{V}}{{\mathrm{G}}_{{\mathrm{jm}}}}+{\mathrm{D}}{{\mathrm{G}}_{{\mathrm{lm}}}}+{\text{ SV}}{{\mathrm{D}}_{{\mathrm{ijl}}}}+{\text{ SV}}{{\mathrm{G}}_{{\mathrm{ijm}}}}+{\text{ SD}}{{\mathrm{G}}_{{\mathrm{ilm}}}}+{\mathrm{VD}}{{\mathrm{G}}_{{\mathrm{jlm}}}}+ \hfill \\ {\mathrm{SVD}}{{\mathrm{G}}_{{\mathrm{ijlm}}}}\,+\,{{\mathrm{e}}_{{\mathrm{ijklm}}}} \hfill \\ \end{gathered} $$

where y_ijklm_ is the response variable in the ith growth system, jth vernalization temperature, kth block, lth depth, and mth genotype; µ is the overall mean; S_i_ is the main effect of the growth system with two levels (SB and NB); V_j_ is the main effect of the vernalization temperature with two levels (NTV and HTV); β_k(ij)_ is the main effect of the block nested in the ith growth system and jth vernalization temperature; D_l_ is the main effect of the lth depth with two levels (SP and DP); δ* is an individual split plot error for each of the main plots (δ_ik_, δ_jk_, δ_lk_, δ_ijk_, δ_ilk_, δ_jlk_, δ_ijlk_ ); G_m_ is the main effect of the mth genotype with twelve levels; SV_ij_, SD_il_, VD_jl_, SG_im_, VG_jm_, and DG_lm_ are the two-way interactions; SVD_ijl_, SVG_ijm_, SDG_ilm_, and VDG_jlm_ are the three-way interactions; SVDG_ijlm_ is the four-way interaction between the growth system (S), vernalization temperature (V), depth (D) and genotype (G); and ε_ijklm_ is the residual error. Here, δ^*^ ~ N (0, $\:{\sigma\:}_{s*}^{2}$), ε_ijklm_ ~ N (0, $\:{\sigma\:}_{e}^{2}$) and cov($\:{\sigma\:}_{e}^{2},{\sigma\:}_{s*}^{2}$) are zero. In the model, all the factors were fitted as fixed except for the split plot error and residual error terms. Orthogonal contrasts were used after the F test for all traits to perform mean comparisons. The variance explained by each factor was estimated via Model (1), but all the factors were used as random effects. All the analyses were performed in the *lme4* [31] and *emmeans* packages [32] in R statistical software version 4.4.2.

## Results

### Variance components

**Fig. 1:**
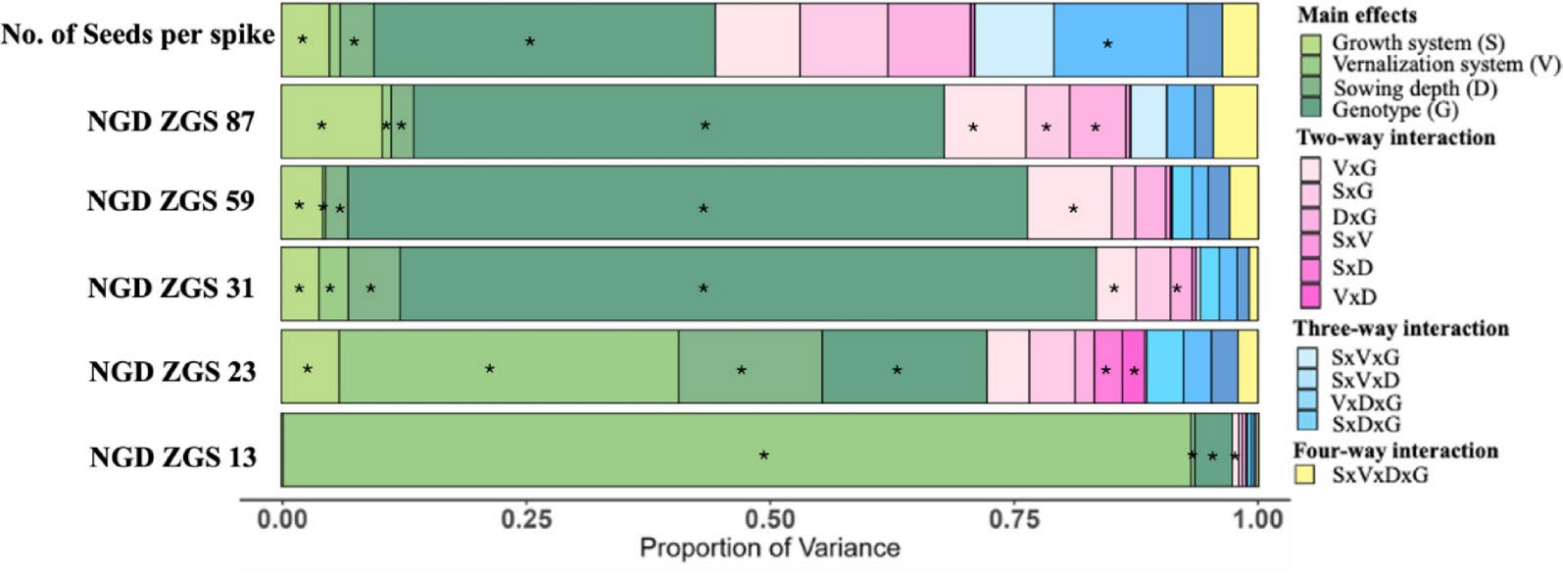
Proportion of the variance explained by the growth system, vernalization temperature, sowing depth, genotype, and their interactions for each phenological stage recorded (ZGS 13, ZGS 23, ZGS 31, ZGS 59, ZGS 87) and for the number of seeds per spike. The main effects or interactions with a star (*) indicate a significant effect of the interaction different from zero, following an F-test at the 5% level of significance

The growth system, vernalization, sowing depth, and genotype had significant effects on the time at which the different phenological stages were reached (Fig. 1). We also detected some significant interactions between the growth system, vernalization, sowing depth, and genotype (Fig. 1).

### Early growth development

On average, SB reached the three-leaf (ZGS 13) and tillering stages (ZGS 23) 1 day earlier than NB did and reached the first node stage five days earlier (ZGS 31, Table S1, Table S2).

In terms of sowing depth, the SP reached the three-leaf stage one day earlier than the DP did (ZGS 13, Table S1), and the SP reached the first node stage four days earlier than the DP did (ZGS 31, Table S2). Although significant interactions between the growth system and sowing depth as well as vernalization system and sowing depth were observed at the tillering stage (ZGS 23; Fig. 1), these interactions were due mainly to magnitude differences rather than changes in rank (Table S1).

**Fig. 2:**
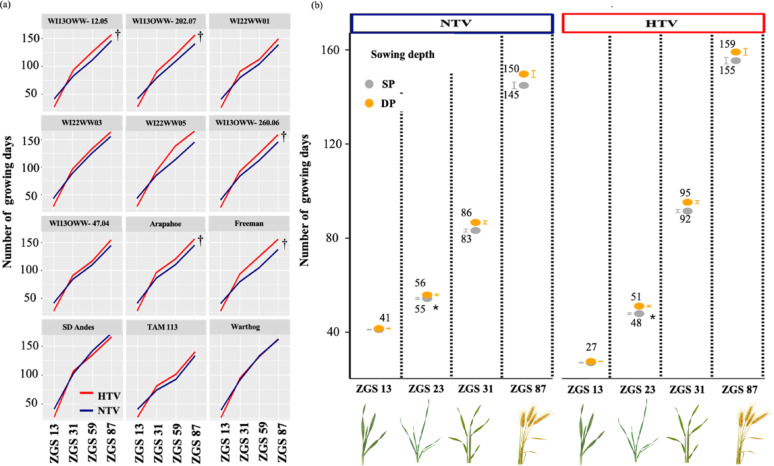
Genotypic responses to vernalization temperature and sowing depth. Phenology in two vernalization treatments: NTV (blue) and HTV (red) for (a) each genotype, † denotes a significant difference in a genotype at two different vernalization system (NTV and HTV) for ZGS 87 following a contrast, with a F-test at the 5% level of significance, and (b) response to sowing depth, a star (*) indicates a significant effect of the interaction different from zero, following an F-test at the 5% level of significance

In the case of the vernalization system, HTV occurred earlier than NTV did for all initial growth stages (14 days for ZGS 13 and 5 days for ZGS 23) (Table S1). However, at the first node stage (ZGS 31), plants in NTV presented a significantly shorter cycle for all the genotypes except for SD Andes and Warthog, where no differences among the vernalization treatments were found (Fig. 2B; Table S2). Finally, although significant interactions were detected between the growth system and vernalization system at the first node stage (ZGS 31; Fig. 1), these interactions differed mainly in magnitude (Tables S2).

### Heading and physiological maturity stages

Heading was reached on average 12 days earlier in SB than in NB (Table S2). Speed breeding was also faster than NB at the physiological maturity stage (ZGS 87) for all the genotypes except TAM 113, which was not significantly different between the systems (Table S3). In terms of planting depth, SP was on average six days earlier than DP to reach heading (ZGS 59, Table S2) and five days earlier to reach maturity (Table S3).

Although a significant interaction between genotype and vernalization system was found for the number of growing days to reach heading (ZGS 59) and maturity (ZGS 87, Fig. 1), the interaction effects were mainly magnitude differences, with most genotypes growing a few days later under HTV than under NTV, while others were not different (Table S3). Genotypes WI13OWW-12.05, WI13OWW-260.06, WI22WW05, and Freeman presented significant differences between vernalization systems at heading (Table S2), whereas no significant differences were detected between NTV and HTV for the remaining genotypes (Table S2). Furthermore, genotypes WI13OWW-12.05, WI13OWW-220.07, WI13OWW-260.06, WI22WW05, Arapahoe, and Freeman presented significant differences between vernalization systems at maturity (Table S4), whereas no significant differences were found between NTV and HTV for the remaining genotypes (Table S4).

### Number of seeds per spike

A significant three-way interaction was found for vernalization temperature, sowing depth, and genotype for thenumber of seeds per spike (Table S3). No difference in seed number was detected between NTV and HTV or between SP and DP, except for one genotype (WI22WW05), which presented significantly less seed numbers at DP under HTV (Table S3).

## Discussion

The present study developed a protocol to apply SB to winter wheat and examine the efficacy of using SB for the generation advancement of diverse populations of hard red winter wheat. Two vernalization systems (NTV and HTV) were tested to explore genotypic responses and the efficacy of using HTV for generational advancement. Finally, to evaluate the effects of sowing depth and their interaction with the vernalization system, two sowing depths (SP: 1 cm and DP: 2.5 cm) were compared.

### System comparison for crop development

Overall, we found that, on average, the SB system was 12 days faster than the NB system to reach heading (ZGS 59) and SB was 17 days earlier than the NB system to reach maturity (ZGS 87). This result coincides with other SB studies in winter wheat, in which differences of 10 [12] and 12 days [23] were found between SB and NB for flowering (ZGS 61). Compared with that in NB, the faster growth rate in SB is due to a longer photoperiod and higher growth temperature; therefore, a faster transition between growth stages occurs [14]. The breeding cycle is reduced by 17 days per generation, 43 days per year using SB, and the first yield phenotypic value can be obtained one year earlier than using NB. We also focused on optimizing vernalization and sowing depth, which reduced the length of the cycle by another five days, but there is room to add other adjustments with the potential to shorten the breeding cycle even further, for example, the use of LED lights with high wavelengths [33], high-density planting [24], or early harvest [34].

### Comparison of the vernalization conditions for crop development

Compared with NTV, HTV accelerated the initial vegetative growth stages (i.e., ZGS 13 and ZGS 23) in both SB and NB. After the plants reached the first node stage (ZGS 31), the effect of HTV slowed compared with that of NTV, resulting in plants in HTV reaching heading later than those in NTV. The faster initial vegetative growth in HTV is explained by a shorter phyllochron in response to the high vernalization temperature (10 °C) and long photoperiod (22 h) [35, 36]. However, NTV resulted in faster transitions between phenological stages after the first node (ZGS 31), and floral initiation started sooner, possibly because the genotype’s vernalization requirement (vernalization saturation) is reached earlier under NTV [37]. Cha et al. [19, 38] argued that higher temperatures during vernalization shorten the length of the crop cycle compared with standard vernalization because of greater accumulated growing degree days. However, this was not observed in all genotypes for the entire growing season in our study. We found that four of the twelve genotypes reached heading (ZGS 59) earlier in NTV, whereas the other eight genotypes did not significantly differ when vernalized via HTV or NTV. These results coincide with previous findings reported by Cha et al. [19] working with European and Australian winter wheat germplasm. Even though Cha et al. [19] tested some winter wheat cultivars originating in the United States, this sample was not representative of the germplasm grown in the Upper Midwest (generally characterized by a higher vernalization requirement). It was necessary to test the protocol to evaluate its efficacy in other germplasm. Additionally, vernalization optimization was necessary to adapt the combination of HTV–SB to a breeding context, where large and diverse populations are advanced, and all genotypes must flower to avoid unintended selection pressure. In this study, diverse sets of genotypes were chosen with different SNP variants for several markers at the VRN genes (Table S1), ensuring the representation of genotypes with different vernalization requirements. Additionally, a comparison with NTV was needed to understand the differences in the response to the vernalization systems. In our study, all the genotypes headed when HTV or NTV was used. This indicates the efficacy of using HTV even for genotypes with diverse vernalization requirements. On average, the NTVs and HTVs took 147 and 157 days to harvest, respectively. Considering the difference in time between the two vernalizing conditions and the simplicity and potential to carry large breeding populations in high-throughput systems, our HTV is an option to be utilized in combination with SB for fast generational advancement.

### Sowing depth

We hypothesized that the use of SP could help shorten the growing cycle of winter wheat. The SP was, on average, six days faster than the DP for days to heading (ZGS 59) and five days faster for maturity (ZGS 87). Therefore, using SP could be an option for further reducing the length of the growing cycle in the generational advancement process. The SB–HTV–SP combination has a growing cycle with 150 growing days to reach maturity (ZGS 87), which allows for a reduction of the breeding cycle by 30 days per year. Because plants were staked in our experiment, one aspect that we could not evaluate and would be worth studying further is the possible negative effect of shallow planting on plant lodging.

### Number of seeds per Spike

**Fig. 3:**
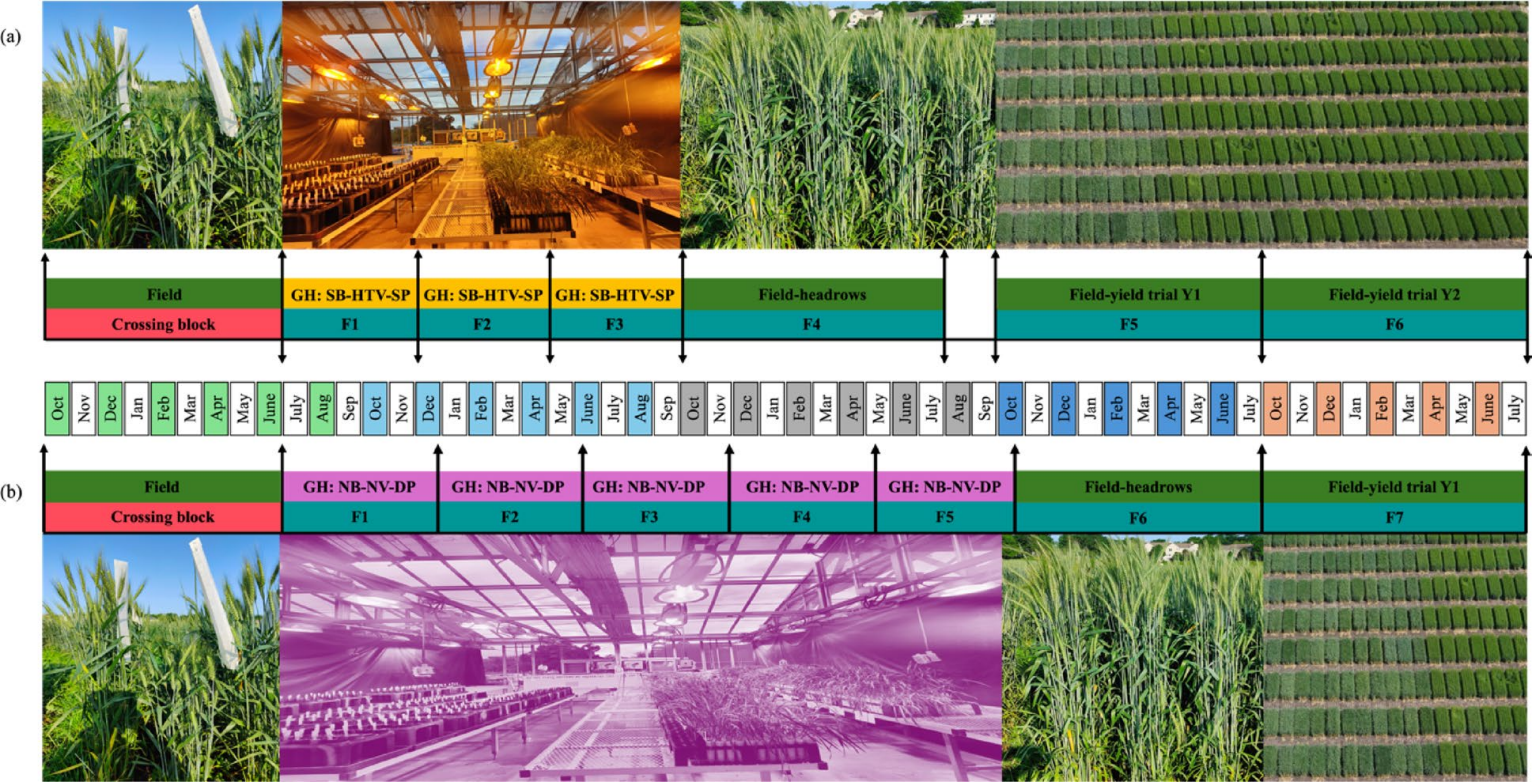
Schematic timeline from crossing to generational advancement yield trials in the field following (a) speed breeding- high-throughput vernalization-shallow planting (SB-HTV-SP); Genotype WI22WW03 was used as a reference for estimating the timeframe required for each SB-HTV-SP cycle, (b) normal breeding- normal vernalization- deep planting (NB-NTV-DP system) in the pipeline; Genotype SD Andes was used as a reference for estimating the timeframe required for each NB-NV-DP cycle

When the single-seed descent (SSD) method is utilized in SB for generation advancement, only a few seeds are required for advancing to the next generation cycle [34, 39]. In our study, a single spike from each plant was harvested to determine if we could obtain enough seeds for the next generation. Genotypes significantly differed in the number of seeds when plants were grown under different vernalizing conditions or sowing depths; however, in all cases, the number of seeds was greater than necessary for advancement. On average, the genotypes had a minimum of approximately 40 seeds from each spike for all conditions tested. Therefore, with our protocol, which combines SB, HTV, and SP, we will obtain enough seeds to multiply the next generation in the greenhouse or transition to a head-to-row scheme in a conventional field nursery using the available seed quantity, making it a comprehensive scheme (Fig. 3a).

### Practical implications

Plant breeding requires the constant generation of genetic diversity [40], and therefore, in winter wheat breeding, populations are diverse in terms of multiple traits, including vernalization requirements. To apply SB in winter wheat breeding, it is therefore necessary to optimize vernalization under SB for diverse populations where all genotypes need to flower. There is diversity in responses to reach vernalization, but it is a period that all winter wheat cultivars should pass through [19]. It also has a direct influence on the length of the growing cycle in winter wheat. Optimizing vernalization conditions to shorten the length of the breeding cycle is needed to increase the genetic gain per unit of time via SB [14, 41]. A shorter breeding cycle enhances the impact of other available tools, such as phenomics [42], genomics [43], training population optimization [44], genotype by environment modeling [45], multi-environment design [46, 47], and selection for the target environment and market use [48, 49]. The ability to accelerate the breeding process using available greenhouses to advance large breeding populations by combining SB with HTV will allow us to reduce the generational time to respond quickly to changes in demand [2], increase farm yields [3, 4, 50] and update the high-value market requirements in the Midwest of the US [48].

Using the developed protocol, we can reduce the time required for both parent’s selection as well as cultivar development [51]. Because of the unsuitability of using the SB system for crossing, crossing is usually conducted in the field. We use early harvest (late June) as soon as the F_1_ seed reaches physiological maturity to protect it from being lost by weather, rodents, or pests, as well as advance it as soon as possible. Following that, F_1_to F_3_ could be advancedin the SB-HTV-SP system in 15 months (Fig. 3). This would allow the next filial generation, F_4,_ to be grown as heads to rows in the field, obtaining the first phenotypes for those lines and those selected, producing enough seeds to start grain yield trials in F_5_ (Fig. 3). Phenotypic data for diseases, maturity, height, and agronomics collected at F_4_ will be combined with phenotypic and grain yield data from F_5_ trials and integrated into the genomic prediction training population to select promising cultivars to assign to multi-environment trials and parents to start a new breeding cycle. Similarly, yield trials could be done in the following generations for 2–3 years and eventually cultivar release in 7–8 years in comparison to 13–14 years using conventional field system (Fig. 3A). When using NB-NTV-DP system for generational advancement, assuming that procedures before and after the greenhouse generational advance are the same, it would not be possible to plant F_4_ from the greenhouse in the field in the same season because they would be ready after the planting window for winter wheat in the Midwest [52]. The most viable option is to continue the generational advance in the greenhouse, allowing us to advance two extra generations (F_4_, F_5_), harvesting the F_6_ seeds in a period of 27 months. The F_6_ generation will then be planted in the next field season as a head-to-row, and yield trials could be conducted at F_7_ when enough seed is produced. When compared to the SB-HTV-SP system, NB-NTV-DP requires one extra calendar year, or one extra field season, and it is two filial generations behind from getting field and yield evaluation, increasing the generational time between crosses, and to select parents to start a new breeding cycle and for cultivar development (Fig. 3B).

## Conclusion

The study revealed a significant reduction in the time required to reach phenological stages when SB was used for generational advancement in hard red winter wheat genotypes. The study also tested the use of two vernalizing conditions (HTV and NTV) and two sowing depths (SP and DP) to develop an efficient protocol for winter wheat that would ensure vernalization for all genotypes. On average, NTV was ten days faster than HTV at the time of harvest (ZGS 87). Therefore, HTV remains an option for breeding programs, as it can be utilized in the greenhouse itself, offering a higher throughput pipeline than growth chambers, which are generally limited in space. Similarly, we found a difference of five days when sowing at SP compared with DP. Therefore, SP has the advantage of reducing the number of days needed to reach the harvest stage and can be used in combination with HTV to reduce the cycle length.

## Supplementary Information

Below is the link to the electronic supplementary material.

Supplementary Material 1.

## Data Availability

The data used and/or analyzed in the current study is available through the Figshare repository, which is available at[https://figshare.com/s/f44d56aaeb8cc45a6151](https:/figshare.com/s/f44d56aaeb8cc45a6151).

## Abbreviations

BLUE: Best linear unbiased predictor
DP: Deep planting
HTV: High-throughput vernalization
NGD: Number of growing days
NTV: Normal treatment vernalization
NB: Normal growth conditions
SP: Shallow planting
SB: Speed breeding
ZGS: Zadok growth stage
