# Supplementary material for: Optimized protocol for high-throughput vernalization with speed breeding in winter wheat
Source: Plant Methods. 2025 Dec 1;21:156. doi: 10.1186/s13007-025-01473-7 (PMC12690896; doi:10.1186/s13007-025-01473-7)
Supplement: Supplementary file 1 — Supplementary Material 1. [file 13007_2025_1473_MOESM1_ESM.docx]

Optimized protocol for high-throughput vernalization with speed breeding in winter wheat

Rishap Dhakal ^1^, Pablo Sandro ^1^, Lucía Gutiérrez ^2^

Affiliations: ^1^Department of Plant and Agroecosystem Sciences, University of Wisconsin-Madison, 1575 Linden Drive, Madison, WI 53706, USA

^2^ Department of Plant Breeding, Swedish University of Agricultural Sciences (SLU), SE-230 53, Alnarp, Sweden

Corresponding Author: gutierrezcha@wisc.edu

# **Supplementary figure and tables**


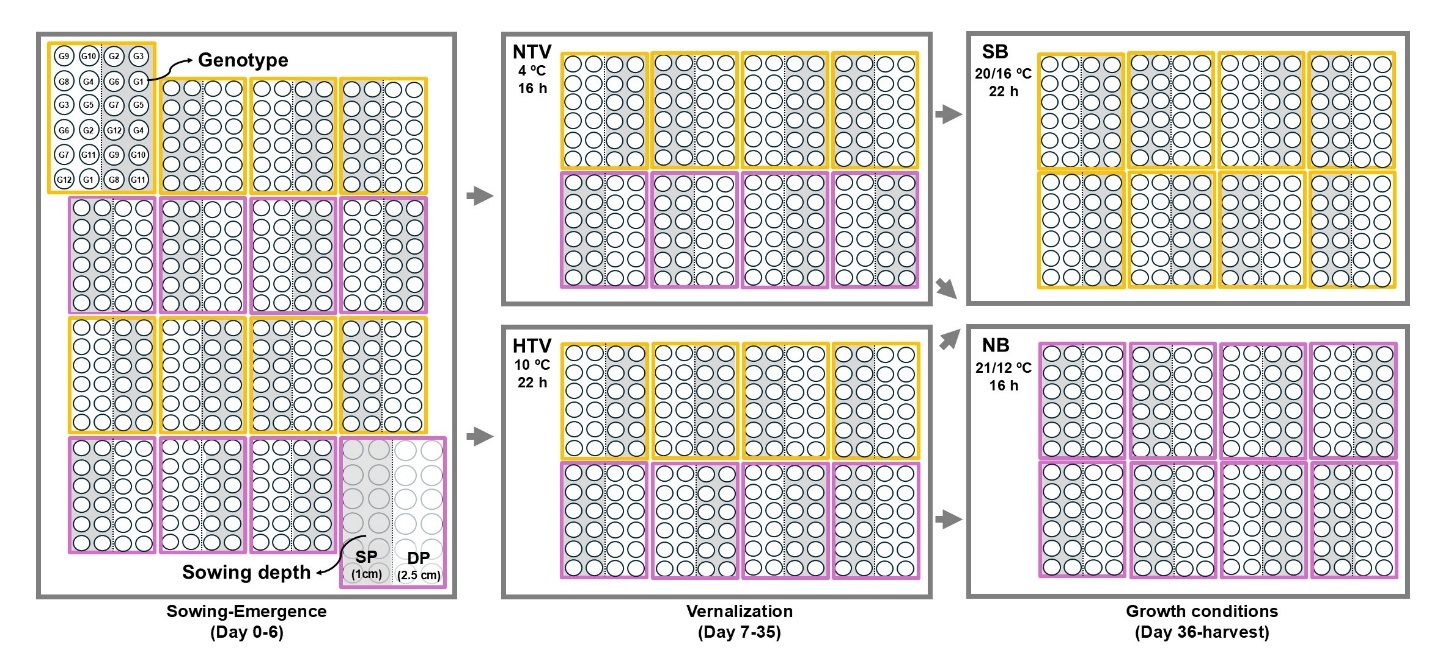


**Figure S1.** Schematic flowchart of treatments applied in the study from sowing to final growth conditions. SP, shallow plating; DP, deep planting; NTV, normal treatment vernalization; HTV, high throughput vernalization; SB, speed breeding growth condition; NB, normal breeding growth condition. Trays are sorted in this diagram for illustration purposes but were fully randomized within systems at each step.


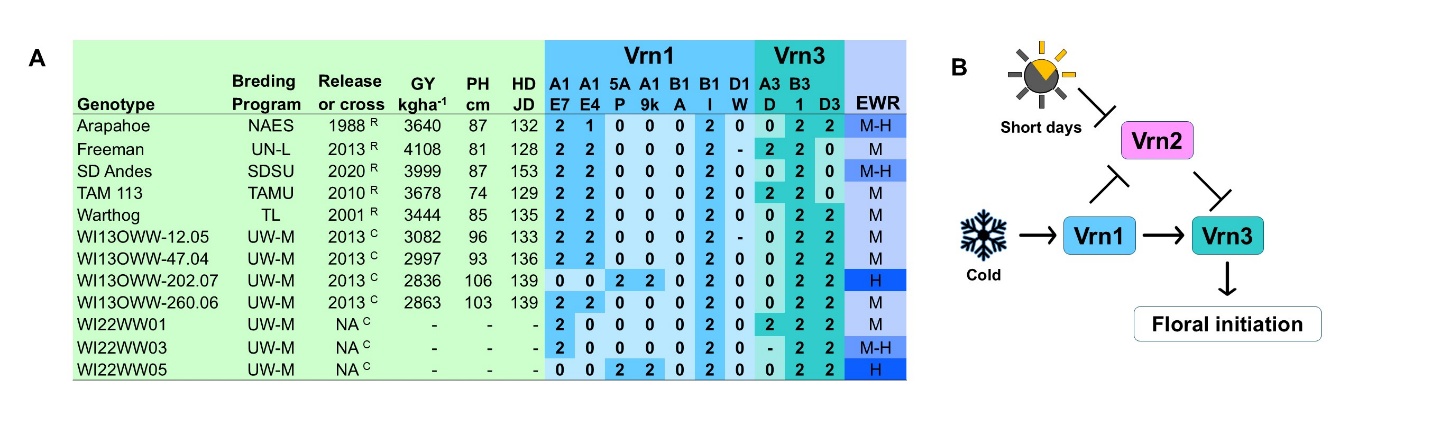


**Figure S2.** (a) Characterization of twelve hard red winter wheat genotypes evaluated in the study along with marker SNPs for the VRN genes in the A, B and D sub genomes of chromosomes 5 and 7. GY, grain yield; HD, heading date; JD, Julian date; and PH, plant height based on historical information from yield trials in the U.S. from data retrieved from T3/wheat CAP database ([https://wheatcap.triticeaetoolbox.org](https://wheatcap.triticeaetoolbox.org/), accessed 25 Nov 2024). Breeding program abbreviations: NAES, Nebraska Agricultural Experiment Station; SDSU, South Dakota State University; TAMU, Texas A and M University; TL, Thompson Limited; UN-L, University of Nebraska-Lincoln; UW-M, University of Wisconsin-Madison. For Release or Cross, R corresponds to the year of release and C represents the year when the cross was made. The ***Vrn1*** genes characterized were: A1_E7 VRN-A1_EXON7, A1_E4 VRN-A1_EXON4, VRN 5AP VRN 5Aprom.K.38, and VRN A19k Vrn-A1_9K0001 on chromosome 5A, VRN B1A VRN-B1_AGS2000 and VRN B1I VRN-B1_I_INS on chromosome 5B, and VRN D1W VRN-D1_W on chromosome 5D. The ***Vrn3*** genes characterized were: VRN A3D VRN NCB_A3Del, VRN B31 VRN-B3_1, and VRN D3 VRND3 on chromosomes 7A, 7B and 7D respectively. (b) A simplified floral induction pathway for winter wheat adapted from Yan et al., 2006. *The floral induction pathway in wheat is primarily regulated by three genes:* ***Vrn1, Vrn2, and Vrn3****.* ***Vrn1*** *and* ***Vrn3*** *act as positive regulators of flowering, whereas* ***Vrn2*** *functions as a repressor. Exposure to prolonged periods of cold temperature increases the transcript levels of Vrn1 and Vrn3, while short-day photoperiods suppress Vrn2 expression. Vrn1 is located on chromosomes* ***5A, 5B, and 5D****, and Vrn3 is located on* ***7A, 7B, and 7D****. The vernalization requirement is genotype-dependent and determined by the presence of dominant or recessive alleles at Vrn loci across the sub-genomes. In Figure S2(a), SNP markers for Vrn1 and Vrn3 are shown to illustrate the genetic diversity of the material used in this study. Genotypes were classified for* ***expected vernalization requirement (EVR)*** *based on their allelic composition at* ***VRN1*** *and* ***VRN3*** *loci. Each dominant allele (coded as 2) reduces vernalization requirement, while each recessive allele (0) increases it. Genotypes carrying most or all recessive alleles across VRN1 and VRN3 loci, resulting in a* ***low dominant allele count****, such as* ***WI22WW05*** *or* ***WI13OWW-202.07*** *were classified as* ***High EVR.*** *Genotypes with a mixture of dominant and recessive alleles such as and* ***SD Andes****,* ***Arapahoe****, and* ***WI22WW03*** *were classified as* ***Medium–High EVR****. The remaining genotypes, with a* ***higher number of dominant alleles****, were classified as* ***Medium EVR****. The final classification for each genotype is provided on the table.*

**Table S1. Growing days BLUEs for three leaf stage (ZGS 13 and tillering stage (ZGS 23) for genotype by growth system, vernalization system, and sowing depth. † indicates significant two way interaction present for NGD at ZGS 13 and NGD at ZGS 23.**

| **ZGS 13** † | | | | | | | | | | | | | | |
| --- | --- | --- | --- | --- | --- | --- | --- | --- | --- | --- | --- | --- | --- | --- |
|  | **SB** | | | | | | | **NB** | | | | | | |
|  | **HTV** | | | | **NTV** | | | | **HTV** | | | | **NTV** | |
| **Genotype** | **SP** | | **DP** | | **SP** | | **DP** | | **SP** | | **DP** | | **SP** | **DP** |
| Arapahoe | 26 | | 27 | | 40 | | 41 | | 28 | | 28 | | 41 | 42 |
| Freeman | 26 | | 28 | | 41 | | 41 | | 27 | | 28 | | 40 | 41 |
| SD Andes | 26 | | 27 | | 41 | | 42 | | 28 | | 28 | | 41 | 42 |
| TAM 113 | 26 | | 28 | | 42 | | 42 | | 27 | | 29 | | 40 | 41 |
| Warthog | 26 | | 26 | | 39 | | 41 | | 28 | | 27 | | 40 | 39 |
| WI13OWW-12.05 | 26 | | 27 | | 42 | | 42 | | 28 | | 28 | | 41 | 42 |
| WI13OWW-47.04 | 27 | | 27 | | 40 | | 42 | | 27 | | 28 | | 42 | 42 |
| WI13OWW-202.07 | 27 | | 27 | | 42 | | 42 | | 28 | | 29 | | 42 | 43 |
| WI13OWW260.06 | 26 | | 26 | | 41 | | 41 | | 29 | | 27 | | 40 | 40 |
| WI22WW01 | 26 | | 26 | | 41 | | 42 | | 27 | | 27 | | 42 | 41 |
| WI22WW03 | 28 | | 30 | | 43 | | 43 | | 28 | | 29 | | 43 | 43 |
| WI22WW05 | 27 | | 28 | | 43 | | 43 | | 29 | | 30 | | 43 | 44 |
| Mean | 26.4 | | 27.5 | | 41.2 | | 41.8 | | 27.8 | | 28.1 | | 41.2 | 41.6 |
| SD | 0.6 | | 1.1 | | 1.2 | | 0.7 | | 0.7 | | 0.9 | | 1.1 | 1.3 |
| **ZGS 23** † | | | | | | | | | | | | | | |
|  | **SB** | | | | | | | **NB** | | | | | | |
|  | **HTV** | | | **NTV** | | | | **HTV** | | | | **NTV** | | |
| **Genotype** | **SP** | **DP** | | **SP** | | **DP** | | **SP** | | **DP** | | **SP** | | **DP** |
| Arapahoe | 46 | | 49 | | 53 | | 55 | | 46 | | 51 | | 55 | 58 |
| Freeman | 49 | | 52 | | 55 | | 58 | | 53 | | 52 | | 55 | 56 |
| SD Andes | 46 | | 49 | | 52 | | 54 | | 48 | | 51 | | 56 | 58 |
| TAM 113 | 46 | | 51 | | 52 | | 55 | | 49 | | 50 | | 54 | 56 |
| Warthog | 46 | | 50 | | 51 | | 55 | | 49 | | 49 | | 55 | 54 |
| WI13OWW-12.05 | 46 | | 51 | | 55 | | 56 | | 50 | | 52 | | 56 | 58 |
| WI13OWW-47.04 | 47 | | 55 | | 53 | | 56 | | 51 | | 51 | | 56 | 58 |
| WI13OWW-202.07 | 47 | | 55 | | 54 | | 55 | | 50 | | 52 | | 57 | 55 |
| WI13OWW260.06 | 45 | | 51 | | 54 | | 54 | | 49 | | 51 | | 55 | 54 |
| WI22WW01 | 44 | | 49 | | 52 | | 53 | | 46 | | 46 | | 55 | 55 |
| WI22WW03 | 50 | | 59 | | 54 | | 58 | | 47 | | 52 | | 57 | 57 |
| WI22WW05 | 47 | | 47 | | 55 | | 59 | | 52 | | 55 | | 57 | 59 |
| Mean | 46.5 | | 51.5 | | 53.3 | | 55.6 | | 49.1 | | 51 | | 55.6 | 56.5 |
| SD | 1.6 | | 3.3 | | 1.3 | | 1.8 | | 2.2 | | 2.1 | | 0.9 | 1.7 |

The growing days are shown for each genotype, growth system (speed breeding, SB or normal breeding, NB), vernalization condition (high-throughput vernalization, HTV or normal treatment vernalization, NTV), and sowing depth (deep planting, DP or shallow planting, SP).

**Table S2. Growing days BLUEs for first node (ZGS 31) and heading stage (ZGS 59) for genotype by growth system, vernalization system, and sowing depth. † Indicates significant two way interaction present for NGD at ZGS 31 and NGD at ZGS 59.**

| **ZGS 31** † | | | | | | | | | | | | | | |
| --- | --- | --- | --- | --- | --- | --- | --- | --- | --- | --- | --- | --- | --- | --- |
|  | **SB** | | | | | | | **NB** | | | | | | |
|  | **HTV** | | | | **NTV** | | | | **HTV** | | | | **NTV** | |
| **Genotype** | **SP** | | **DP** | | **SP** | | **DP** | | **SP** | | **DP** | | **SP** | **DP** |
| Arapahoe | 89 | | 99 | | 78 | | 91 | | 98 | | 100 | | 84 | 92 |
| Freeman | 88 | | 95 | | 76 | | 76 | | 94 | | 97 | | 82 | 85 |
| SD Andes | 102 | | 107 | | 101 | | 102 | | 105 | | 113 | | 99 | 107 |
| TAM 113 | 75 | | 79 | | 69 | | 71 | | 88 | | 85 | | 77 | 80 |
| Warthog | 90 | | 99 | | 86 | | 91 | | 96 | | 97 | | 96 | 97 |
| WI13OWW-12.05 | 91 | | 93 | | 81 | | 80 | | 95 | | 95 | | 85 | 86 |
| WI13OWW-47.04 | 89 | | 88 | | 78 | | 78 | | 91 | | 96 | | 87 | 93 |
| WI13OWW-202.07 | 90 | | 96 | | 76 | | 77 | | 91 | | 87 | | 81 | 85 |
| WI13OWW260.06 | 88 | | 94 | | 80 | | 83 | | 94 | | 94 | | 85 | 87 |
| WI22WW01 | 87 | | 92 | | 75 | | 81 | | 93 | | 94 | | 82 | 84 |
| WI22WW03 | 96 | | 103 | | 84 | | 91 | | 94 | | 95 | | 86 | 96 |
| WI22WW05 | 86 | | 94 | | 78 | | 82 | | 92 | | 99 | | 93 | 88 |
| Mean | 89.2 | | 94.1 | | 80.1 | | 83.5 | | 94.2 | | 96.0 | | 86.4 | 90 |
| SD | 6.2 | | 7.1 | | 7.8 | | 8.6 | | 4.2 | | 6.9 | | 6.4 | 7.3 |
| **ZGS 59** † | | | | | | | | | | | | | | |
|  | **SB** | | | | | | | **NB** | | | | | | |
|  | **HTV** | | | **NTV** | | | | **HTV** | | | | **NTV** | | |
| **Genotype** | **SP** | **DP** | | **SP** | | **DP** | | **SP** | | **DP** | | **SP** | | **DP** |
| Arapahoe | 110 | | 117 | | 95 | | 104 | | 113 | | 129 | | 113 | 129 |
| Freeman | 109 | | 126 | | 100 | | 94 | | 110 | | 115 | | 110 | 115 |
| SD Andes | 120 | | 133 | | 126 | | 143 | | 151 | | 149 | | 151 | 149 |
| TAM 113 | 92 | | 99 | | 86 | | 89 | | 92 | | 105 | | 92 | 105 |
| Warthog | 123 | | 125 | | 124 | | 122 | | 131 | | 157 | | 131 | 157 |
| WI13OWW-12.05 | 110 | | 120 | | 106 | | 99 | | 115 | | 127 | | 115 | 127 |
| WI13OWW-47.04 | 113 | | 107 | | 98 | | 100 | | 118 | | 123 | | 118 | 123 |
| WI13OWW-202.07 | 116 | | 110 | | 101 | | 98 | | 117 | | 123 | | 117 | 123 |
| WI13OWW260.06 | 118 | | 118 | | 102 | | 110 | | 119 | | 116 | | 119 | 116 |
| WI22WW01 | 108 | | 105 | | 94 | | 103 | | 111 | | 110 | | 111 | 110 |
| WI22WW03 | 128 | | 138 | | 111 | | 121 | | 122 | | 146 | | 122 | 146 |
| WI22WW05 | 123 | | 127 | | 101 | | 110 | | 122 | | 122 | | 122 | 122 |
| Mean | 114.1 | | 118.7 | | 103.6 | | 107.7 | | 118.4 | | 126.8 | | 118.4 | 126.8 |
| SD | 9.4 | | 11.8 | | 11.7 | | 14.8 | | 13.8 | | 16.0 | | 13.8 | 16.0 |

The growing days are shown for each genotype, growth system (speed breeding, SB or normal breeding, NB), vernalization condition (high-throughput vernalization, HTV or normal treatment vernalization, NV), and sowing depth (deep planting, DP or shallow planting, SP).

**Table S3. Growing days BLUEs for maturity (ZGS 87) and number of seeds per spike for genotype by growth system, vernalization system, and sowing depth. † indicates significant two way interaction present for NGD at ZGS 87 and ‡ indicate significant three way interaction present for number of seeds per spike.**

| **ZGS 87** † | | | | | | | | | | | | | | |
| --- | --- | --- | --- | --- | --- | --- | --- | --- | --- | --- | --- | --- | --- | --- |
|  | **SB** | | | | | | | **NB** | | | | | | |
|  | **HTV** | | | | **NTV** | | | | **HTV** | | | | **NTV** | |
| **Genotype** | **SP** | | **DP** | | **SP** | | **DP** | | **SP** | | **DP** | | **SP** | **DP** |
| Arapahoe | 144 | | 150 | | 127 | | 137 | | 163 | | 168 | | 148 | 168 |
| Freeman | 140 | | 154 | | 133 | | 127 | | 161 | | 169 | | 144 | 146 |
| SD Andes | 149 | | 158 | | 158 | | 174 | | 170 | | 188 | | 180 | 178 |
| TAM 113 | 133 | | 135 | | 131 | | 127 | | 149 | | 144 | | 136 | 140 |
| Warthog | 154 | | 156 | | 141 | | 155 | | 166 | | 176 | | 165 | 187 |
| WI13OWW-12.05 | 146 | | 152 | | 140 | | 132 | | 171 | | 160 | | 148 | 165 |
| WI13OWW-47.04 | 150 | | 140 | | 128 | | 133 | | 164 | | 166 | | 158 | 160 |
| WI13OWW-202.07 | 148 | | 149 | | 140 | | 131 | | 166 | | 163 | | 149 | 142 |
| WI13OWW260.06 | 151 | | 152 | | 136 | | 146 | | 170 | | 163 | | 153 | 148 |
| WI22WW01 | 141 | | 146 | | 127 | | 133 | | 154 | | 158 | | 148 | 148 |
| WI22WW03 | 156 | | 164 | | 146 | | 148 | | 163 | | 173 | | 156 | 175 |
| WI22WW05 | 152 | | 150 | | 135 | | 142 | | 172 | | 188 | | 152 | 153 |
| Mean | 147 | | 150.5 | | 136.8 | | 140.4 | | 164.0 | | 168.0 | | 153.0 | 159.1 |
| SD | 6.5 | | 7.7 | | 9.0 | | 13.7 | | 6.9 | | 12.3 | | 11.1 | 15.4 |
| **No. of seeds per spike** ‡ | | | | | | | | | | | | | | |
|  | **SB** | | | | | | | **NB** | | | | | | |
|  | **HTV** | | | **NTV** | | | | **HTV** | | | | **NTV** | | |
| **Genotype** | **SP** | **DP** | | **SP** | | **DP** | | **SP** | | **DP** | | **SP** | | **DP** |
| Arapahoe | 47 | | 41 | | 43 | | 41 | | 38 | | 28 | | 42 | 28 |
| Freeman | 58 | | 36 | | 63 | | 51 | | 45 | | 45 | | 55 | 40 |
| SD Andes | 45 | | 39 | | 39 | | 42 | | 45 | | 19 | | 12 | 26 |
| TAM 113 | 37 | | 54 | | 42 | | 28 | | 31 | | 57 | | 50 | 38 |
| Warthog | 38 | | 41 | | 36 | | 38 | | 25 | | 23 | | 25 | 9 |
| WI13OWW-12.05 | 65 | | 52 | | 48 | | 47 | | 41 | | 31 | | 51 | 44 |
| WI13OWW-47.04 | 43 | | 57 | | 53 | | 53 | | 34 | | 55 | | 37 | 31 |
| WI13OWW-202.07 | 50 | | 47 | | 41 | | 54 | | 39 | | 35 | | 46 | 47 |
| WI13OWW260.06 | 53 | | 56 | | 49 | | 41 | | 35 | | 43 | | 44 | 45 |
| WI22WW01 | 52 | | 58 | | 53 | | 48 | | 60 | | 45 | | 67 | 65 |
| WI22WW03 | 44 | | 45 | | 58 | | 49 | | 57 | | 38 | | 47 | 24 |
| WI22WW05 | 55 | | 31 | | 59 | | 60 | | 51 | | 6 | | 50 | 54 |
| Mean | 48.9 | | 46.4 | | 48.6 | | 46 | | 41.7 | | 35.4 | | 43.8 | 37.5 |
| SD | 8.2 | | 8.9 | | 8.6 | | 8.5 | | 10.4 | | 14.9 | | 14.2 | 15.0 |

The growing days are shown for each genotype, growth system (speed breeding, SB or normal breeding, NB), vernalization condition (high-throughput vernalization, HTV or normal treatment vernalization, NTV), and sowing depth (deep planting, DP or shallow planting, SP).
